# 2,6-Dibromo-4-(2-hy­droxy­eth­yl)phenol

**DOI:** 10.1107/S1600536811046538

**Published:** 2011-11-12

**Authors:** Ding-qiang Lu, Hong Chai, Xiu-quan Ling, Jia Chen, Jia-li Wang

**Affiliations:** a State Key Laboratory of Materials-Oriented Chemical Engineering, School of Pharmaceutical Sciences, Nanjing University of Technology, Xinmofan Road No. 5 Nanjing, Nanjing 210009, People’s Republic of China; b State Key Laboratory of Materials-Oriented Chemical Engineering, College of Life Science and Pharmaceutical Engineering, Nanjing University of Technology, Xinmofan Road No. 5 Nanjing, Nanjing 210009, People’s Republic of China

## Abstract

The title compound, C_8_H_8_Br_2_O_2_, crystallized with two independent mol­ecules (*A* and *B*) in the asymmetric unit. They differ in the conformation of the 2-hy­droxy­ethyl chain with the C—C—C—O torsion angle being −68.0 (12)° in mol­ecule *A* and 172.2 (9)° in mol­ecule *B*. In the crystal, the *A* mol­ecules are linked *via* pairs of O—H⋯O hydrogen bonds, forming inversion dimers, while the *B* mol­ecules are linked *via* an O—H⋯O hydrogen bond, forming a polymeric chain propagating in [010]. In addition, there are O—H⋯O and O—H⋯Br hydrogen bonds, and Br⋯Br [3.599 (2) Å] and π–π inter­actions [centroid–centroid distances = 3.581 (6) and 3.931 (6) Å], leading to the formation of a two-dimensional network parallel to (001).

## Related literature

For background and further synthetic details, see: Guerard *et al.* (2009 ▶); Bovicelli *et al.* (2007 ▶). For standard bond-length data, see: Allen *et al.* (1987 ▶). For a related structure, see: Zhu *et al.* (2011 ▶)
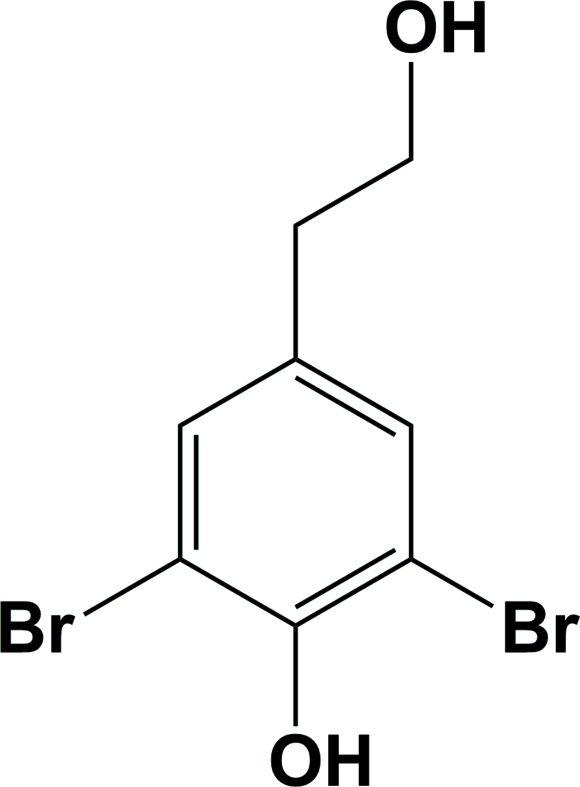


## Experimental

### 

#### Crystal data

C_8_H_8_Br_2_O_2_
*M* *_r_* = 295.94
Triclinic, 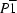

*a* = 8.5740 (17) Å
*b* = 9.845 (2) Å
*c* = 11.392 (2) Å
α = 86.08 (3)°
β = 75.79 (3)°
γ = 87.39 (3)°
*V* = 929.6 (3) Å^3^
*Z* = 4
Mo *K*α radiation
μ = 8.68 mm^−1^
*T* = 293 K
0.20 × 0.10 × 0.10 mm

#### Data collection

Enraf–Nonius CAD-4 diffractometer
Absorption correction: ψ scan (North *et al.*, 1968 ▶) *T*_min_ = 0.276, *T*_max_ = 0.478
3664 measured reflections
3416 independent reflections
1874 reflections with *I* > 2σ(*I*)
*R*_int_ = 0.087
3 standard reflections every 200 reflections  intensity decay: 1%

#### Refinement

*R*[*F* ^2^ > 2σ(*F* ^2^)] = 0.067
*wR*(*F* ^2^) = 0.161
*S* = 1.00
3416 reflections
217 parameters
1 restraint
H-atom parameters constrained
Δρ_max_ = 0.63 e Å^−3^
Δρ_min_ = −0.72 e Å^−3^

### 

Data collection: *CAD-4 EXPRESS* (Enraf–Nonius, 1994 ▶); cell refinement: *CAD-4 EXPRESS*; data reduction: *XCAD4* (Harms & Wocadlo, 1995 ▶); program(s) used to solve structure: *SHELXS97* (Sheldrick, 2008 ▶); program(s) used to refine structure: *SHELXL97* (Sheldrick, 2008 ▶); molecular graphics: *SHELXTL* (Sheldrick, 2008 ▶) and *Mercury* (Macrae *et al.*, 2008 ▶); software used to prepare material for publication: *PLATON* (Spek, 2009 ▶) and *publCIF* (Westrip, 2010 ▶).

## Supplementary Material

Crystal structure: contains datablock(s) global, I. DOI: 10.1107/S1600536811046538/su2342sup1.cif

Structure factors: contains datablock(s) I. DOI: 10.1107/S1600536811046538/su2342Isup2.hkl

Supplementary material file. DOI: 10.1107/S1600536811046538/su2342Isup3.cml

Additional supplementary materials:  crystallographic information; 3D view; checkCIF report

## Figures and Tables

**Table 1:** Hydrogen-bond geometry (Å, °)

*D*—H⋯*A*	*D*—H	H⋯*A*	*D*⋯*A*	*D*—H⋯*A*
O1—H1*O*⋯O2^i^	0.82	1.93	2.665 (11)	149
O4—H4*O*⋯O3^ii^	0.85	2.09	2.837 (9)	146
O2—H2*O*⋯O4^iii^	0.82	2.09	2.896 (10)	168
O3—H3*O*⋯Br2^iv^	0.85	2.55	3.291 (7)	147

Symmetry codes: (i) 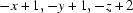; (ii) 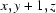; (iii) 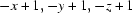; (iv) 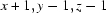.

**Fig. 1.**
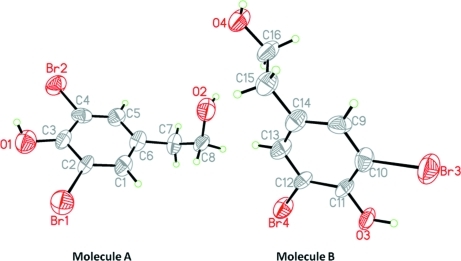
The molecular structure of the two indpendent molecules (A and B) of the title compound, with the atom numbering scheme and displacement ellipsoids drawn at 30% probability levels.

**Fig. 2.**
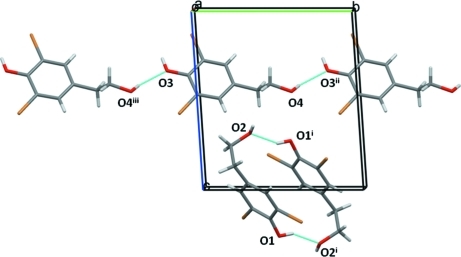
A view along the a axis of the hydrogen bonded inversion dimers of A molecules and the polymer chain of B molecules of the title compound [hydrogen bonds are shown as dashed cyan lines; see Table 1 for details; symmetry codes: (i) -x + 1, -y + 1, -z + 2; (ii) x, y + 1, z; (iii) x, y - 1, z].

**Fig. 3.**
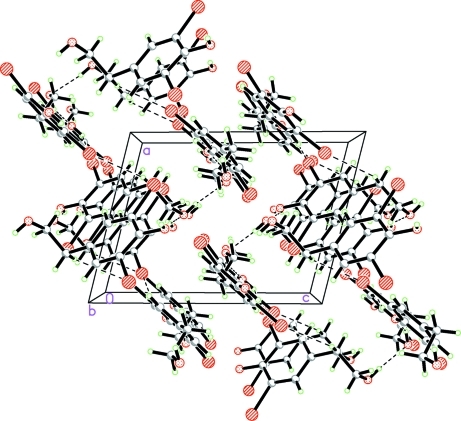
A perspective view along the b axis of the crystal packing of the title compound, showing the O—H···O and O—H···Br hydrogen bonds as dashed lines (see Table 1 for details).

## supplementary crystallographic information

### Comment

The title compound is used as a key intermediate in drug synthesis, and has been synthesized following a procedure described previously by (Bovicelli *et al.*, 2007). We report herein on its synthesis and crystal structure.

The title compound crystallized with two independent molecules (A & B) in the asymmetric unit (Fig. 1). They differ significantly in conformation, as may be seen from the torsion angles in the 2-hydroxyethyl chain. For molecule A the torsion angles C1—C6—C7—C8 and C6—C7—C8—O2 are -56.6 (14) and -68.0 (13)°, respectively, while the corresponding torsion angles C13—C14—C15—C16 and C14—C15—C16—O4 in molecule B are -82.6 (12) and 172.2 (9)°, respectively. The bond lengths (Allen *et al.*, 1987) and bond angles are otherwise within normal ranges.

In the crystal, the A molecules are linked *via* O—H···O hydrogen bonds to form inversion dimers, while the B molecules are linked *via* an O—H···O hydrogen bond to form a polymer chain propagating in [010], see Fig. 2. In the crystal, there are other O—H···O and O—H···Br hydrogen bonds (Table 1), and weak π···π stacking interactions involving the aromatic rings with their inversion related rings. The centroid-centroid distances are 3.931 (6) Å for *Cg*1···*Cg*1^i^ [*Cg*1 is the centroid of ring (C1—C6); symmetry code (i) -*x* + 1, -*y* + 1, -*z* + 2] and 3.581 (6) Å for *Cg*2···*Cg*2^ii^ [*Cg*2 is the centroid of ring (C9—C14); symmetry code (ii) -*x* + 2, -*y*, -*z* + 1]. There is also a short Br1···Br4^iii^ interaction present [3.599 (2) Å; symmetry code: (iii) -*x* + 1, -*y*, -*z* + 2]. These interactions result in the formation of two-dimensional networks lieing parallel to the *ab* plane (Fig. 3).

### Experimental

The title compound was synthesized using a slightly modified version of the procedure reported on by (Bovicelli *et al.*, 2007), using twice the quantity of NaBr. To a solution of 4-hydroxyphenethyl alcohol (217.4 mmol, 30 g) and NaBr (434.8 mmol, 44.34 g) in acetone (600 ml), a solution of oxone (200 g) in water (1 *L*) was added dropwise at 263 K within 3 h. The progress of the reaction was monitored by thin-layer chromatography (TLC, hexane/ethyl acetate 3:2), and when the reaction was over (complete consumption of the substrate), AcOEt (500 ml) was added to the mixture. The organic layer was separated, and the aqueous phase was extracted with two 300 mL portions of AcOEt (300 ml). The combined organic solutions were washed with water (300 ml), dried over anhydrous Na_2_SO_4_(white power,100 g), and evaporated. The product obtained in almost quantitative yield (59.7 g), appeared to be spectroscopically pure: white solid. Crystals of the title compound, suitable for X-ray diffraction analysis, were obtained by slow evaporation of an acetone solution at room temperature.

### Refinement

H atoms were positioned geometrically and constrained to ride on their parent atoms: O—H = 0.82 or 0.85 Å, C—H = 0.93, 0.98 and 0.97 Å for aromatic, methine and methylene H atoms, respectively, with *U*_iso_(H) = k × *U*_eq_(C), where k = 1.5 for methyl H atoms, and k = 1.2 for all other H atoms.

### Crystal data

**Table:**

C_8_H_8_Br_2_O_2_	*Z* = 4
*M**_r_* = 295.94	*F*(000) = 568
Triclinic, *P*1	*D*_x_ = 2.115 Mg m^−^^3^
Hall symbol: -P 1	Mo *K*α radiation, λ = 0.71073 Å
*a* = 8.5740 (17) Å	Cell parameters from 25 reflections
*b* = 9.845 (2) Å	θ = 10–13°
*c* = 11.392 (2) Å	µ = 8.68 mm^−^^1^
α = 86.08 (3)°	*T* = 293 K
β = 75.79 (3)°	Block, colourless
γ = 87.39 (3)°	0.20 × 0.10 × 0.10 mm
*V* = 929.6 (3) Å^3^	

### Data collection

**Table:**

Enraf–Nonius CAD-4 diffractometer	1874 reflections with *I* > 2σ(*I*)
Radiation source: fine-focus sealed tube	*R*_int_ = 0.087
graphite	θ_max_ = 25.4°, θ_min_ = 1.9°
ω/2θ scans	*h* = 0→10
Absorption correction: ψ scan (North *et al.*, 1968)	*k* = −11→11
*T*_min_ = 0.276, *T*_max_ = 0.478	*l* = −13→13
3664 measured reflections	3 standard reflections every 200 reflections
3416 independent reflections	intensity decay: 1%

### Refinement

**Table:**

Refinement on *F*^2^	Primary atom site location: structure-invariant direct methods
Least-squares matrix: full	Secondary atom site location: difference Fourier map
*R*[*F*^2^ > 2σ(*F*^2^)] = 0.067	Hydrogen site location: inferred from neighbouring sites
*wR*(*F*^2^) = 0.161	H-atom parameters constrained
*S* = 1.00	*w* = 1/[σ^2^(*F*_o_^2^) + (0.072*P*)^2^] where *P* = (*F*_o_^2^ + 2*F*_c_^2^)/3
3416 reflections	(Δ/σ)_max_ < 0.001
217 parameters	Δρ_max_ = 0.63 e Å^−^^3^
1 restraint	Δρ_min_ = −0.72 e Å^−^^3^

### Special details

**Table:**

Geometry. Bond distances, angles *etc*. have been calculated using the rounded fractional coordinates. All su's are estimated from the variances of the (full) variance-covariance matrix. The cell e.s.d.'s are taken into account in the estimation of distances, angles and torsion angles
Refinement. Refinement of *F*^2^ against ALL reflections. The weighted *R*-factor *wR* and goodness of fit *S* are based on *F*^2^, conventional *R*-factors *R* are based on *F*, with *F* set to zero for negative *F*^2^. The threshold expression of *F*^2^ > σ(*F*^2^) is used only for calculating *R*-factors(gt) *etc*. and is not relevant to the choice of reflections for refinement. *R*-factors based on *F*^2^ are statistically about twice as large as those based on *F*, and *R*- factors based on ALL data will be even larger.

### Fractional atomic coordinates and isotropic or equivalent isotropic displacement parameters (Å^2^)

**Table:**

	*x*	*y*	*z*	*U*_iso_*/*U*_eq_	
Br1	0.68665 (14)	0.22580 (14)	1.16796 (12)	0.0661 (5)	
Br2	0.15646 (15)	0.59523 (13)	1.18207 (12)	0.0703 (5)	
O1	0.4403 (9)	0.4472 (8)	1.2658 (7)	0.070 (3)	
O2	0.4818 (9)	0.3052 (8)	0.6910 (7)	0.066 (3)	
C1	0.4707 (12)	0.2251 (11)	1.0171 (9)	0.050 (4)	
C2	0.5035 (11)	0.2892 (9)	1.1125 (9)	0.043 (3)	
C3	0.4101 (13)	0.3990 (10)	1.1676 (9)	0.049 (4)	
C4	0.2875 (12)	0.4483 (10)	1.1133 (9)	0.050 (3)	
C5	0.2490 (12)	0.3861 (10)	1.0200 (9)	0.050 (4)	
C6	0.3404 (13)	0.2735 (11)	0.9689 (10)	0.053 (4)	
C7	0.2967 (12)	0.1977 (12)	0.8703 (9)	0.056 (4)	
C8	0.4333 (12)	0.1821 (9)	0.7602 (9)	0.049 (3)	
Br3	1.12544 (15)	−0.02904 (13)	0.15184 (12)	0.0742 (5)	
Br4	0.64211 (13)	−0.12778 (11)	0.58132 (10)	0.0558 (4)	
O3	0.8985 (8)	−0.1939 (6)	0.3477 (6)	0.049 (2)	
O4	0.7207 (8)	0.5766 (7)	0.4611 (6)	0.056 (3)	
C9	0.9903 (11)	0.1713 (10)	0.3173 (9)	0.046 (4)	
C10	0.9906 (11)	0.0338 (10)	0.2919 (9)	0.045 (3)	
C11	0.8926 (11)	−0.0623 (9)	0.3728 (9)	0.039 (3)	
C12	0.7878 (10)	−0.0124 (9)	0.4787 (9)	0.036 (3)	
C13	0.7884 (11)	0.1234 (10)	0.5006 (10)	0.048 (3)	
C14	0.8884 (11)	0.2154 (10)	0.4232 (10)	0.047 (3)	
C15	0.8832 (12)	0.3638 (11)	0.4564 (10)	0.052 (4)	
C16	0.7456 (13)	0.4450 (10)	0.4198 (11)	0.059 (4)	
H1	0.53400	0.15080	0.98510	0.0600*	
H1O	0.46340	0.52760	1.25190	0.1050*	
H2O	0.41630	0.32850	0.65120	0.0990*	
H5	0.16160	0.41900	0.99050	0.0600*	
H7A	0.26060	0.10790	0.90340	0.0670*	
H7B	0.20760	0.24600	0.84580	0.0670*	
H8A	0.52520	0.14080	0.78600	0.0580*	
H8B	0.40200	0.11990	0.70820	0.0580*	
H3O	0.96250	−0.21940	0.28290	0.0590*	
H4O	0.80520	0.62110	0.43080	0.0840*	
H9	1.05730	0.23190	0.26410	0.0560*	
H13	0.71880	0.15470	0.57020	0.0580*	
H15A	0.87040	0.36660	0.54320	0.0620*	
H15B	0.98440	0.40530	0.41600	0.0620*	
H16A	0.64730	0.39530	0.45080	0.0710*	
H16C	0.76670	0.45120	0.33200	0.0710*	

### Atomic displacement parameters (Å^2^)

**Table:**

	*U*^11^	*U*^22^	*U*^33^	*U*^12^	*U*^13^	*U*^23^
Br1	0.0559 (7)	0.0740 (9)	0.0707 (8)	0.0040 (6)	−0.0190 (6)	−0.0112 (6)
Br2	0.0754 (9)	0.0556 (8)	0.0731 (9)	0.0157 (6)	−0.0043 (6)	−0.0185 (6)
O1	0.079 (6)	0.067 (6)	0.062 (5)	−0.009 (4)	−0.010 (4)	−0.010 (4)
O2	0.074 (5)	0.066 (5)	0.056 (5)	−0.011 (4)	−0.010 (4)	−0.015 (4)
C1	0.043 (6)	0.055 (7)	0.041 (6)	−0.001 (5)	0.013 (5)	−0.012 (5)
C2	0.045 (6)	0.034 (5)	0.050 (6)	−0.003 (4)	−0.009 (5)	−0.016 (4)
C3	0.062 (7)	0.038 (6)	0.047 (6)	−0.007 (5)	−0.007 (5)	−0.016 (5)
C4	0.049 (6)	0.045 (6)	0.046 (6)	0.006 (5)	0.003 (5)	0.011 (5)
C5	0.042 (6)	0.051 (7)	0.052 (7)	0.008 (5)	−0.003 (5)	−0.010 (5)
C6	0.049 (6)	0.054 (7)	0.057 (7)	−0.003 (5)	−0.010 (5)	−0.018 (5)
C7	0.052 (6)	0.065 (8)	0.049 (7)	−0.004 (6)	−0.003 (5)	−0.023 (5)
C8	0.062 (7)	0.032 (5)	0.051 (6)	−0.002 (5)	−0.009 (5)	−0.012 (4)
Br3	0.0710 (8)	0.0624 (8)	0.0702 (9)	0.0004 (6)	0.0217 (6)	−0.0141 (6)
Br4	0.0524 (7)	0.0416 (6)	0.0638 (7)	−0.0090 (5)	0.0075 (5)	−0.0094 (5)
O3	0.055 (4)	0.037 (4)	0.046 (4)	0.004 (3)	0.008 (3)	−0.016 (3)
O4	0.056 (5)	0.044 (4)	0.065 (5)	0.000 (4)	−0.004 (4)	−0.017 (4)
C9	0.040 (6)	0.035 (6)	0.056 (7)	0.000 (5)	0.000 (5)	0.011 (5)
C10	0.038 (5)	0.048 (6)	0.046 (6)	−0.007 (5)	0.001 (4)	−0.012 (5)
C11	0.043 (5)	0.024 (5)	0.051 (6)	0.002 (4)	−0.013 (4)	−0.017 (4)
C12	0.033 (5)	0.024 (5)	0.051 (6)	−0.001 (4)	−0.007 (4)	−0.010 (4)
C13	0.030 (5)	0.045 (6)	0.063 (7)	0.010 (5)	0.002 (5)	−0.012 (5)
C14	0.039 (5)	0.045 (5)	0.061 (7)	0.003 (5)	−0.016 (5)	−0.015 (5)
C15	0.043 (6)	0.052 (6)	0.062 (7)	0.005 (5)	−0.012 (5)	−0.015 (5)
C16	0.061 (7)	0.038 (6)	0.071 (8)	0.007 (5)	−0.002 (6)	−0.016 (5)

### Geometric parameters (Å, °)

**Table:**

Br1—C2	1.897 (10)	C1—H1	0.9300
Br2—C4	1.882 (10)	C5—H5	0.9300
Br3—C10	1.852 (10)	C7—H7B	0.9700
Br4—C12	1.857 (9)	C7—H7A	0.9700
O1—C3	1.330 (13)	C8—H8B	0.9700
O2—C8	1.420 (12)	C8—H8A	0.9700
O1—H1O	0.8200	C9—C10	1.403 (14)
O2—H2O	0.8200	C9—C14	1.388 (15)
O3—C11	1.342 (11)	C10—C11	1.423 (14)
O4—C16	1.398 (12)	C11—C12	1.419 (14)
O3—H3O	0.8500	C12—C13	1.377 (13)
O4—H4O	0.8500	C13—C14	1.387 (15)
C1—C2	1.385 (14)	C14—C15	1.530 (15)
C1—C6	1.412 (15)	C15—C16	1.522 (16)
C2—C3	1.404 (14)	C9—H9	0.9300
C3—C4	1.398 (15)	C13—H13	0.9300
C4—C5	1.376 (14)	C15—H15A	0.9700
C5—C6	1.403 (15)	C15—H15B	0.9700
C6—C7	1.518 (15)	C16—H16A	0.9700
C7—C8	1.502 (14)	C16—H16C	0.9700
			
C3—O1—H1O	109.00	C7—C8—H8B	109.00
C8—O2—H2O	109.00	O2—C8—H8A	109.00
C11—O3—H3O	119.00	C10—C9—C14	118.9 (9)
C16—O4—H4O	108.00	C9—C10—C11	122.0 (9)
C2—C1—C6	119.4 (10)	Br3—C10—C11	117.8 (7)
C1—C2—C3	123.3 (9)	Br3—C10—C9	120.2 (7)
Br1—C2—C1	117.5 (7)	O3—C11—C12	122.1 (8)
Br1—C2—C3	119.2 (7)	O3—C11—C10	120.7 (9)
O1—C3—C2	119.7 (10)	C10—C11—C12	117.2 (8)
C2—C3—C4	115.5 (9)	Br4—C12—C13	120.5 (8)
O1—C3—C4	124.8 (9)	Br4—C12—C11	119.9 (7)
Br2—C4—C3	118.1 (7)	C11—C12—C13	119.6 (9)
Br2—C4—C5	118.8 (8)	C12—C13—C14	122.8 (10)
C3—C4—C5	122.9 (9)	C9—C14—C15	121.4 (9)
C4—C5—C6	120.6 (10)	C9—C14—C13	119.5 (9)
C1—C6—C5	118.1 (10)	C13—C14—C15	119.1 (9)
C5—C6—C7	122.1 (10)	C14—C15—C16	111.6 (9)
C1—C6—C7	119.6 (10)	O4—C16—C15	114.7 (9)
C6—C7—C8	113.7 (9)	C10—C9—H9	121.00
O2—C8—C7	115.0 (8)	C14—C9—H9	121.00
C2—C1—H1	120.00	C12—C13—H13	119.00
C6—C1—H1	120.00	C14—C13—H13	119.00
C6—C5—H5	120.00	C14—C15—H15A	109.00
C4—C5—H5	120.00	C14—C15—H15B	109.00
C6—C7—H7A	109.00	C16—C15—H15A	109.00
C8—C7—H7B	109.00	C16—C15—H15B	109.00
C6—C7—H7B	109.00	H15A—C15—H15B	108.00
C8—C7—H7A	109.00	O4—C16—H16A	109.00
H7A—C7—H7B	108.00	O4—C16—H16C	109.00
O2—C8—H8B	109.00	C15—C16—H16A	109.00
C7—C8—H8A	109.00	C15—C16—H16C	109.00
H8A—C8—H8B	108.00	H16A—C16—H16C	108.00
			
C6—C1—C2—Br1	−176.8 (8)	C14—C9—C10—Br3	−179.8 (8)
C6—C1—C2—C3	2.0 (16)	C14—C9—C10—C11	−1.6 (15)
C2—C1—C6—C5	0.2 (15)	C10—C9—C14—C13	−0.7 (15)
C2—C1—C6—C7	−175.8 (9)	C10—C9—C14—C15	179.6 (9)
Br1—C2—C3—O1	−7.3 (13)	Br3—C10—C11—O3	0.0 (13)
Br1—C2—C3—C4	173.7 (7)	Br3—C10—C11—C12	−178.9 (7)
C1—C2—C3—O1	173.9 (10)	C9—C10—C11—O3	−178.2 (9)
C1—C2—C3—C4	−5.1 (15)	C9—C10—C11—C12	2.9 (14)
O1—C3—C4—Br2	0.9 (14)	O3—C11—C12—Br4	−4.8 (13)
O1—C3—C4—C5	−172.6 (10)	O3—C11—C12—C13	179.2 (9)
C2—C3—C4—Br2	179.8 (7)	C10—C11—C12—Br4	174.2 (7)
C2—C3—C4—C5	6.3 (15)	C10—C11—C12—C13	−1.9 (14)
Br2—C4—C5—C6	−177.9 (8)	Br4—C12—C13—C14	−176.3 (8)
C3—C4—C5—C6	−4.5 (16)	C11—C12—C13—C14	−0.2 (15)
C4—C5—C6—C1	0.9 (16)	C12—C13—C14—C9	1.6 (16)
C4—C5—C6—C7	176.8 (10)	C12—C13—C14—C15	−178.7 (9)
C1—C6—C7—C8	−56.6 (13)	C9—C14—C15—C16	97.1 (12)
C5—C6—C7—C8	127.6 (11)	C13—C14—C15—C16	−82.6 (12)
C6—C7—C8—O2	−68.0 (12)	C14—C15—C16—O4	172.2 (9)

### Hydrogen-bond geometry (Å, °)

**Table:**

*D*—H···*A*	*D*—H	H···*A*	*D*···*A*	*D*—H···*A*
O1—H1O···O2^i^	0.82	1.93	2.665 (11)	149
O4—H4O···O3^ii^	0.85	2.09	2.837 (9)	146
O2—H2O···O4^iii^	0.82	2.09	2.896 (10)	168
O3—H3O···Br2^iv^	0.85	2.55	3.291 (7)	147

Symmetry codes: (i) −*x*+1, −*y*+1, −*z*+2; (ii) *x*, *y*+1, *z*; (iii) −*x*+1, −*y*+1, −*z*+1; (iv) *x*+1, *y*−1, *z*−1.
